# False Croup

**Published:** 1890-02-08

**Authors:** 


### FALSE CROUP.

Dr. Harvey B. Bashire contributes to the *New York Medical Record* of December last "a clinical note on chloroform water" for the cure of false croup. He uses a solution consisting of five to ten minims of chloroform to an ounce of water, to which is added a little glycerine "to aid the solubility of the chloroform." A teaspoonful of this is given every half-hour during an attack, and if there is any dyspnœa on the following day, a teaspoonful is given every two hours. This plan Dr. Bashire has pursued very successfully. As so-called false croup is a malady of a nervous spasmodic nature, the treatment recommended above is likely to be temporarily beneficial. But we object to the term false croup, or even to the term spasmodic croup, also to the name croup itself. For the term croup has arisen from the "crouping" noise attending a specific inflammation of some part of the air passages. Croup, however, is so firmly established as a term that it would be difficult to abolish it for a more scientific designation. But certainly croup should not be applied to so-called false croup, or otherwise spasmodic croup. For false croup, or spasmodic croup, is in reality a form of the convulsions of children, which may manifest itself slightly by what nurses term "a catch in the breath," or "child crowing," or it may be marked by suffocative paroxysms, from which occasionally death has resulted. In our experience infants in poor health from constitutional causes are most liable to this malady, especially if they are being reared by hand. It is not unusually connected with, or caused by, the irritation of swollen gums during teething, by glandular enlargements in the neck, by disordered stomach, or by accumulation of fœca, matter in the intestines, or by worms. It is, therefore, evident that the cure of the malady requires something more than anti-spasmodics, even if administered in the shape of chloroform. The constitution must be studied, and any existing cause of the disease must be attacked. Neither should we be disposed to trust altogether to chloroform water for the relief of an attack. We have been accustomed to sprinkle the face with cold water, to rub the spine, to apply a cloth or sponge wrung out of hot and cold water alternately to the throat, and to give a stimulant if the child can swallow. Also to lance the gums if necessary. If the fit has come on after a full meal to excite vomiting, and if these measures do not succeed, to use a hot bath, or to put the feet in hot water if a bath cannot be procured. It is, however, during the intervals between attacks that curative agents are most serviceable, but they must be applied to the cause of the "croup," and not to the croup itself.

